# PPP1R13L drives cervical cancer progression by suppressing p63-mediated PTEN transcription

**DOI:** 10.1007/s00018-025-05598-9

**Published:** 2025-02-27

**Authors:** Anjin Wang, Xuelian Liu, Ziyan Liang, Shijie Yao, Shimeng Wan, Hang Ren, Yang Gao, Hua Wang, Hongbin Cai

**Affiliations:** 1 Department of Gynecological Oncology, Zhongnan Hospital, Wuhan University, Wuhan, People’s Republic of China; 2 Hubei Key Laboratory of Tumor Biological Behaviors, Wuhan, People’s Republic of China; 3 Hubei Cancer Clinical Study Center, Wuhan, People’s Republic of China; 4 Department of Gynecological Oncology, Zhongnan Hospital, 169 Donghu Rd., Wuhan City, China

**Keywords:** PPP1R13L, PTEN, Cervical cancer, Glycolysis, p63

## Abstract

PPP1R13L is a conserved inhibitor of p53, selectively regulating a subset of p53 target genes. Previous studies have reported that PPP1R13L promotes cervical cancer progression, yet its precise mechanism remains unclear and warrants further investigation. In this study, we utilized public databases to reveal the correlation between PPP1R13L and tumor progression pathways. Subsequently, we performed functional assays both in vitro and in xenograft models to assess the impact of PPP1R13L on cervical cancer. Our results demonstrate that PPP1R13L promotes cervical cancer cell proliferation, epithelial-mesenchymal transition, cycle progression, and glycolysis via the PTEN/AKT/mTOR pathway. Mechanistically, PPP1R13L regulates the transcription of PTEN through its Ank-SH3 domain interaction with p53 family, p53 and p63. In 293T cells, p53 originally exhibits significantly higher transcriptional activity than p63. However, in cervical cancer—where E6 continuously degrades p53 and p63 is highly expressed—p63 demonstrates a transcriptional activity for PTEN that is comparable to, or even surpasses, that of p53, depending on E6 expression levels. Additionally, in C33A, an HPV-negative cervical cancer cell line, the p53 R273C mutation causes PPP1R13L to exert an opposite effect, and p63 is shown to be inhibited by PPP1R13L independently of p53. Finally, the response elements of PPP1R13L-regulated p53 family target genes were experimentally validated on p63 for the first time. This provides a sequence basis for the selective regulation of p53 family target genes by PPP1R13L. In summary, we underscore the specificity of the PPP1R13L/p63/PTEN axis in cervical cancer and propose that PPP1R13L holds potential as a therapeutic target for cervical cancer treatment.

**Graphical abstract:**

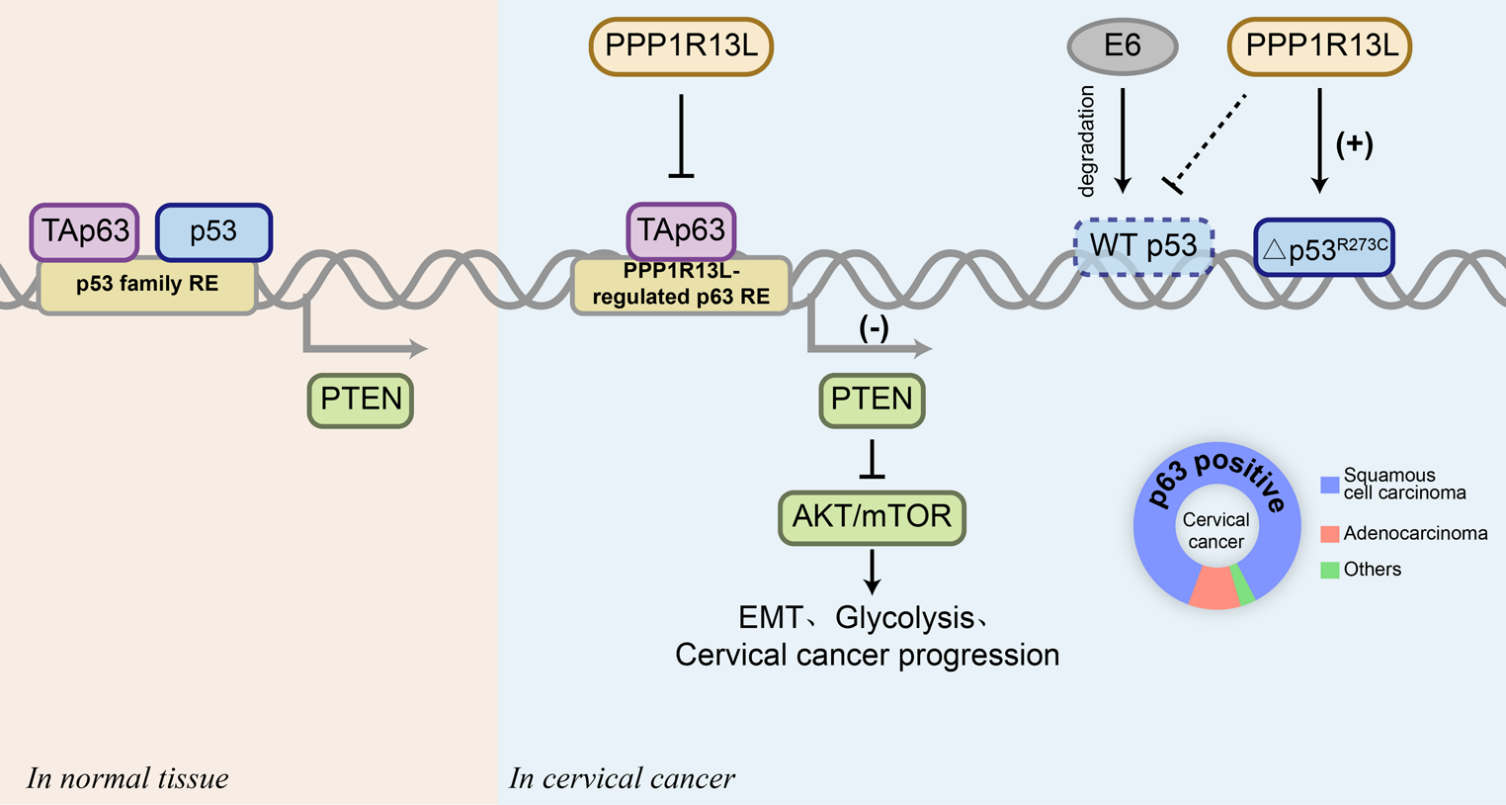

**Supplementary Information:**

The online version contains supplementary material available at 10.1007/s00018-025-05598-9.

## Introduction

Cervical cancer remains a significant global health challenge, particularly in low- and middle-income countries, with 662,301 new cases and 348,874 deaths reported in 2022 [1]. Chronic infection with high-risk human papillomavirus (HPV) subtypes is one of the leading causes of cervical cancer. The squamocolumnar junction (SCJ) is the region where the squamous and glandular cells of the cervix meet, and it is highly susceptible to HPV-induced carcinogenesis [2]. The interaction between the viral E6 protein of HPV and the p53 protein, leading to its degradation, is one of the most critical mechanisms in the pathogenesis of cervical cancer. Consequently, most cervical cancer cells retain a wild-type TP53 gene but exhibit strongly decreased expression of the p53 protein [3].

PPP1R13L (protein phosphatase 1 regulatory subunit 13 like), also known as inhibitor of apoptosis-stimulating protein of p53 (iASPP), was initially identified as a key inhibitor of p53, conserved from worms to humans as an oncoprotein [4]. It can inhibit p53 function by suppressing p53’s transactivation activity on the promoters of pro-apoptotic genes and is overexpressed in various human tumors [5]. It is associated with poor prognosis in multiple cancers, including lung cancer, breast cancer, colorectal cancer, cervical cancer, hepatocellular carcinoma, and gastric cancer [4, 6–10]. Studies on PPP1R13L suggest that it plays roles in apoptosis, senescence, antioxidation, epithelial-mesenchymal transition (EMT), mitotic spindle regulation, immune responses, and other processes, thereby promoting tumor development and chemoresistance, identifying it as a possible diagnostic marker and treatment target in these cancers [5, 11–15].

PTEN (phosphatase and tensin homolog) is a critical tumor suppressor gene, whose protein functions as a lipid phosphatase, dephosphorylating PIP3 into PIP2 and inhibiting the PI3K/AKT signaling pathway. Loss of PTEN is common in cancers, resulting in unrestrained activation of the AKT/mTOR pathway, promoting tumor growth and survival [16]. Additionally, given the significant role of the AKT pathway in glucose metabolism, the loss of PTEN also affects cellular metabolism by driving the Warburg effect, where cancer cells favor glycolysis over aerobic respiration to meet increased energy demands [17].

p63 is crucial for the development of stratified squamous epithelium and is predominantly expressed in basal keratinocytes [18, 19]. In cervical cancer, p63 serves as a marker for differentiating cancer types, with the majority of squamous cell carcinomas exhibiting diffuse nuclear immunoreactivity for this protein [18]. As a member of the p53 family, it has been reported that PPP1R13L selectively inhibits a portion of p63-mediated transcription, similarly to its effects on p53 [20, 21]. However, the focus on the PPP1R13L/p63 interaction and its functions is considerably lower than that on the PPP1R13L/p53.

Here, we identified PPP1R13L as an oncogene associated with the progression of cervical cancer. We demonstrated that PPP1R13L enhances cell proliferation, cell cycle, EMT, and glycolysis in cervical cancer cells by modulating the PTEN/AKT/mTOR pathway. Further investigation revealed that PPP1R13L influences PTEN transcription by interacting with the p53 and p63. Additionally, we discovered that the R273C mutation in p53 causes PPP1R13L to exert an opposite effect on p53. Finally, we confirmed that PPP1R13L inhibits the transcription of PTEN by p63, dependent on specific response elements and emphasized the distinct characteristics of the PPP1R13L/p63/PTEN axis in cervical cancer. In summary, our data indicate that PPP1R13L holds potential in the targeted treatment of cervical cancer.

**Fig. 1:**
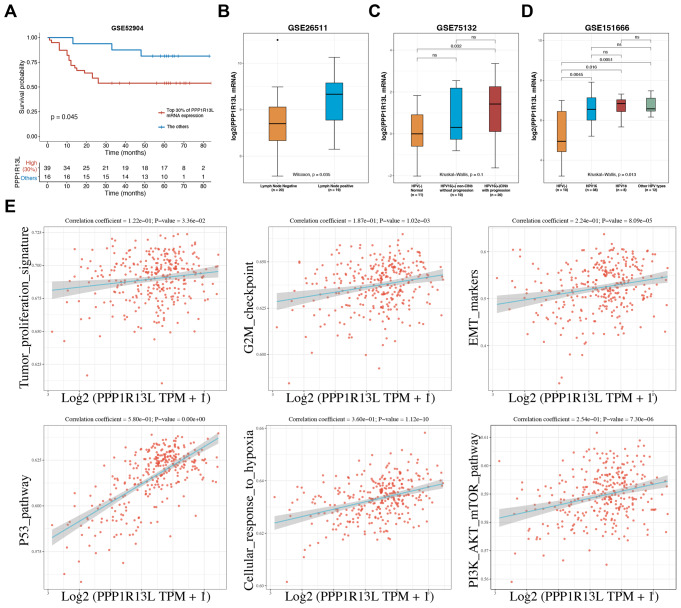
PPP1R13L impacts the prognosis of cervical cancer and reveals the correlation with tumor progression pathways. (**A**) Kaplan-Meier analysis of overall survival in the GSE52904 cohort revealed that the top 30% of patients with the highest *PPP1R13L* mRNA expression had significantly lower survival rates compared to the remaining 70% of patients with lower PPP1R13L expression. (**B**) Analysis of the GSE26511 dataset revealed that cervical cancer patients with lymph node metastasis exhibited higher *PPP1R13L* mRNA expression compared to those without metastasis. (**C**) In the GSE75132 dataset, cervical tissues persistently infected with HPV16 (categorized into non-CIN3 and CIN3) were compared to normal tissues without HPV16 infection. The results showed that *PPP1R13L* mRNA expression progressively increased across these groups. (**D**) In the GSE151666 dataset, HPV-positive cervical tumor tissues exhibited higher *PPP1R13L* mRNA expression compared to HPV-negative primary cervical tumor tissues. Elevated PPP1R13L expression was observed in tumors infected with HPV16, HPV18, and other HPV types (HPV33, 59, 58, 52, 45, 31, 56). (**E**) The ssGSEA analysis of PPP1R13L with tumor proliferation signature, G2M checkpoint, EMT markers, p53 pathway, cellular response to hypoxia, and the PI3K/AKT/mTOR pathway. The data is from TCGA datasets

## Results

### PPP1R13L impacts the prognosis of cervical cancer and reveals the correlation with tumor progression pathways

Based on data from the GSE52904, survival analysis showed that PPP1R13L is associated with poor prognosis in cervical cancer (Fig. 1A). The top 30% of patients with the highest *PPP1R13L* mRNA expression exhibited lower survival rates compared to the 70% of patients with lower PPP1R13L expression. Lymph node involvement is a major prognostic factor in cervical cancer. According to the GSE26511 dataset, patients with lymph node metastasis exhibit higher *PPP1R13L* mRNA expression compared to those without metastasis (Fig. 1B). In GSE75132, cervical tissues persistently infected with HPV16 (categorized into non-CIN3 and CIN3) were compared to normal tissues without HPV16 infection. PPP1R13L expression progressively increased across these groups, with CIN3 tissues showing significantly higher *PPP1R13L* mRNA levels than normal tissues (Fig. 1C). Additionally, according to GSE151666, HPV-positive cervical tumor tissues exhibited higher *PPP1R13L* mRNA expression compared to HPV-negative primary cervical tumor tissues (Fig. 1D). Elevated PPP1R13L expression was observed in HPV16, HPV18, and other types (HPV33, 59, 58, 52, 45, 31, 56). Using ssGSEA analysis (CESC of TCGA data), we showed that PPP1R13L expression is significantly positively correlated with pathways related to tumor proliferation signature, G2M checkpoint, EMT markers, p53 pathway, cellular response to hypoxia, and the PI3K/AKT/mTOR pathway (Fig. 1E).

**Fig. 2:**
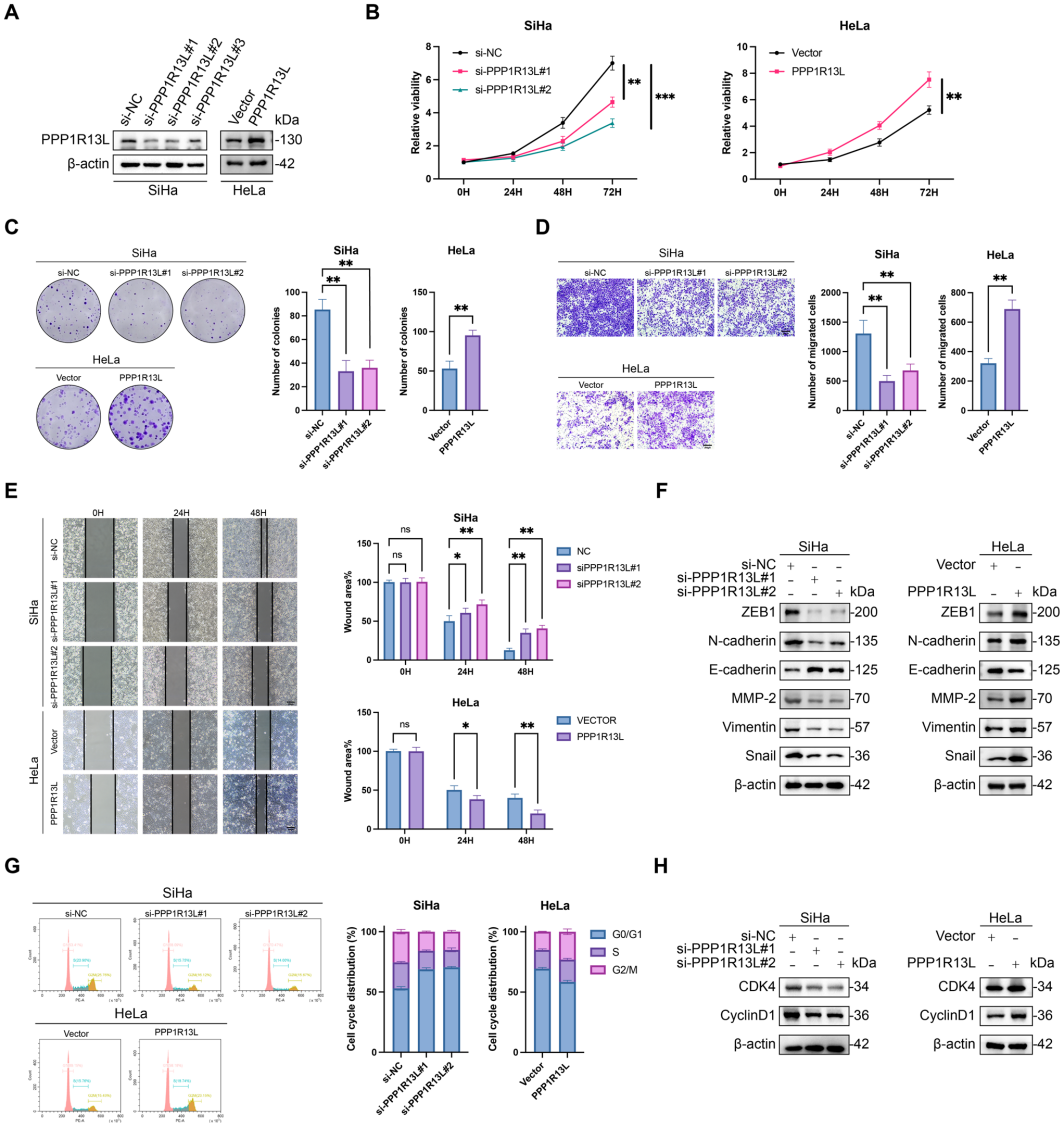
PPP1R13L promotes cervical cancer cell proliferation, cycle progression and facilitates EMT. (**A**) Western blot showed PPP1R13L overexpression in HeLa cells and PPP1R13L knockdown via siRNA in SiHa cells. (**B**,** C**) CCK-8 and colony formation of PPP1R13L-knockdown SiHa cells and PPP1R13L-overexpressing HeLa cells to assess the proliferation capacity. The results indicate that PPP1R13L promotes cervical cancer cell proliferation. (**D**,** E**) Transwell assay and wound-healing assay to detect the migration of PPP1R13L-knockdown SiHa cells and PPP1R13L-overexpressing HeLa cells. The results indicate that PPP1R13L promotes cervical cancer cell migration. Scale bar represents 200 μm. (**F**) The expression of EMT markers (ZEB1, N-cadherin, E‐cadherin, Vimentin, Snail, MMP‐2) in PPP1R13L-knockdown SiHa cells and PPP1R13L-overexpressing HeLa cells. The results indicate that PPP1R13L promotes cervical cancer cell EMT. (**G**) Flow cytometry results indicate that PPP1R13L knockdown in SiHa cells induces cell cycle arrest in the G0/G1 phase, while PPP1R13L overexpression in HeLa cells reduces the proportion of cells in this phase. (**H**) Western blot results show that the expression of cell cycle-related proteins is reduced in PPP1R13L-knockdown SiHa cells and increased in PPP1R13L-overexpressing HeLa cells. *N* = 3 per group. Data are expressed as the mean ± SD. Two-tailed t-tests analyzed two-group differences; one-way ANOVA assessed multiple-group differences. ns, *p* > 0.05; *p < 0.05; **p < 0.01; ***p < 0.001

### PPP1R13L promotes cervical cancer cell proliferation, cycle progression and facilitates EMT

To investigate the role of PPP1R13L in cervical cancer cell lines, we first examined the expression of PPP1R13L in cervical cancer cell lines and tested the knockdown and overexpression efficiency in SiHa and HeLa cells (Fig. S1). We assessed the effects of PPP1R13L overexpression in HeLa cells and PPP1R13L knockdown using siRNA (si-PPP1R13L#1, si-PPP1R13L#2, and si-PPP1R13L#3) in SiHa cells through western blot analysis. To prevent of off-target effects, we selected two highly effective siRNAs, si-PPP1R13L#1 and si-PPP1R13L#2, to independent knockdown of PPP1R13L in subsequent experiments (Fig. 2A).

**Fig. 3:**
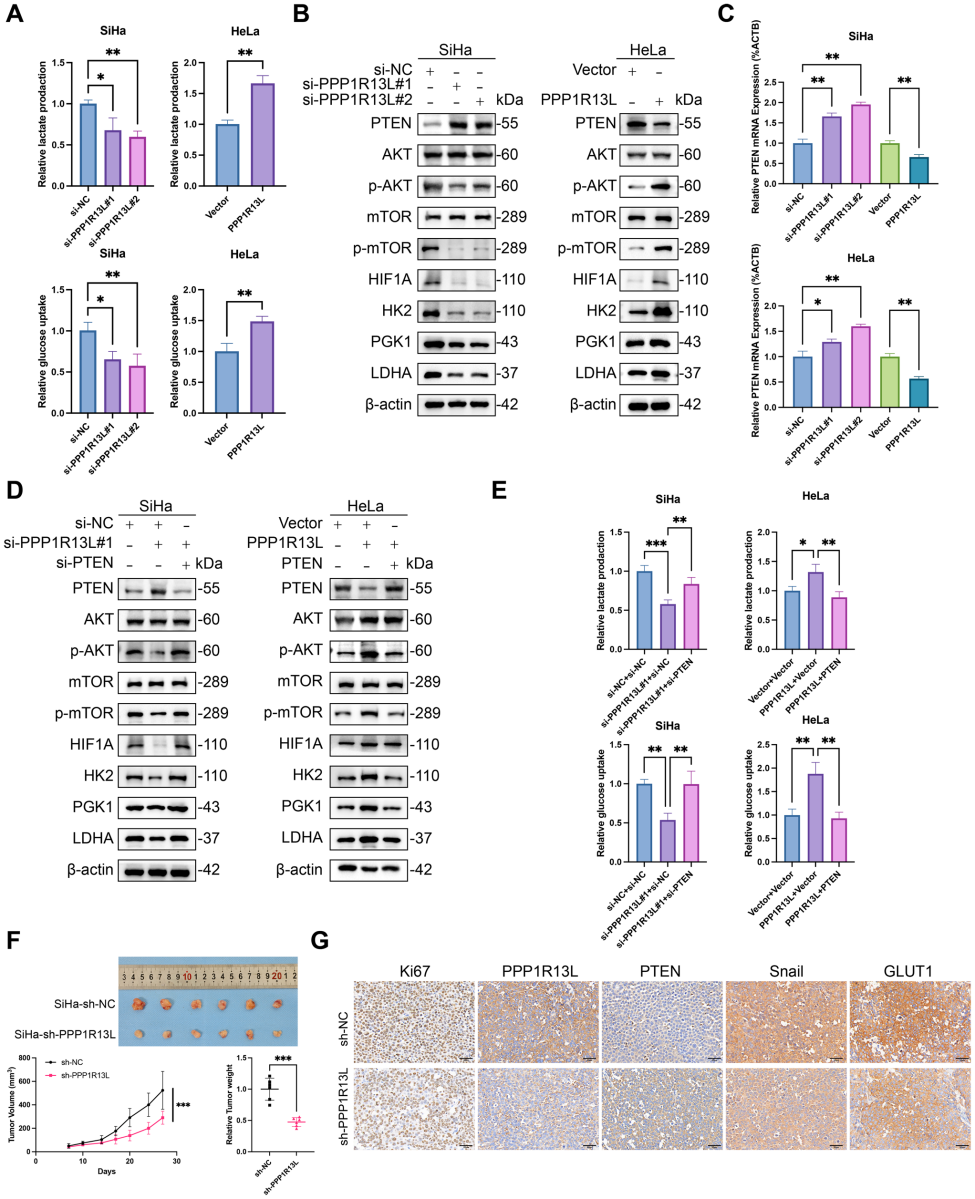
PPP1R13L downregulates PTEN to activate the PI3K/AKT/mTOR pathway and upregulates glycolysis in cervical cancer cells. (**A**) Glucose consumption and lactate production in cervical cancer cells were detected after transfection with siRNAs or PPP1R13L overexpression plasmids for 48 h. Glucose consumption and lactate production were decreased by PPP1R13L knockdown in HeLa cells but increased by PPP1R13L overexpression in SiHa cells. (**B**) Western blot analysis shows that PPP1R13L downregulates PTEN in cervical cancer cells, leading to the activation of the PI3K/AKT/mTOR/HIF1A pathway. Concurrently, several key glycolytic enzymes, including HK2, PGK1, and LDHA, are upregulated. (**C**) The mRNA expression of PTEN after knockdown and overexpression of PPP1R13L in both SiHa and HeLa cells demonstrates that PPP1R13L inhibits *PTEN* mRNA levels in cervical cancer cells. (**D**) The expression of PTEN/AKT/mTOR/HIF1A pathway with key glycolytic enzymes (HK2, PGK1, and LDHA) was detected by western blot. PTEN knockdown reversed the effects of PPP1R13L knockdown in SiHa cells and PTEN overexpression reversed the effects of PPP1R13L overexpression in HeLa cells. (**E**) Glucose consumption and lactate production in cervical cancer cells were detected after transfection for 48 h. PTEN knockdown reversed the effects of PPP1R13L knockdown in SiHa cells and PTEN overexpression reversed the effects of PPP1R13L overexpression in HeLa cells. (**F**) Gross images of xenograft tumors in the SiHa-sh-NC and SiHa-sh-PPP1R13L groups indicate that PPP1R13L inhibits cervical cancer cell growth in vivo, as shown by the reduced tumor volume and weight. *N* = 6 per group. Two-tailed t-tests analyzed two-group differences. *p* > 0.05; *p < 0.05; **p < 0.01; ***p < 0.001. (**G**) IHC of the xenograft tumor model showed that the SiHa-sh-PPP1R13L group exhibited lower Ki67 expression and higher PTEN levels. Moreover, the expression of EMT marker Snail and the glucose transporter GLUT1 was also reduced in the SiHa-sh-PPP1R13L group. Scale bar represents 50 μm. *N* = 3 per group. Data are expressed as the mean ± SD. Two-tailed t-tests analyzed two-group differences; one-way ANOVA assessed multiple-group differences. ns, *p* > 0.05; *p < 0.05; **p < 0.01; ***p < 0.001

With cell counting kit-8 and clonogenic assays, Overexpressing PPP1R13L in HeLa cells promotes cell proliferation, while knocking down PPP1R13L in SiHa cells inhibits it (Fig. 2B, C). For the effect of PPP1R13L on the migration, the results from wound-healing and transwell assays demonstrated that PPP1R13L knockdown inhibited the migration ability of SiHa cells, whereas PPP1R13L overexpression enhanced the it in HeLa cells (Fig. 2D, E).

For the impact of PPP1R13L on EMT, we examined the expression levels of E-cadherin, N-cadherin, ZEB1, Vimentin, Snail, and MMP-2 using western blot. The results revealed that in PPP1R13L-knockdown SiHa cells, E-cadherin expression increased while the levels of N-cadherin, ZEB1, Vimentin, Snail, and MMP-2 decreased. Conversely, in PPP1R13L-overexpressing HeLa cells, the expression of ZEB1, Vimentin, Snail, and MMP-2 was elevated, and E-cadherin expression was reduced (Fig. 2F). Collectively, these changes in EMT markers suggest that PPP1R13L promotes EMT of SiHa and HeLa cells. The findings revealed that PPP1R13L is pivotal in the growth, migration, and EMT of cervical cancer cells, corresponding to Fig. 1E.

Given the correlation between PPP1R13L and the G2/M checkpoint (Fig. 1E), we examined the impact of PPP1R13L on the cell cycle in cervical cancer cells. PPP1R13L knockdown triggered cell cycle arrest in the G0/G1 phase in SiHa cell lines, whereas PPP1R13L overexpression decreased the proportion of cells in this phase (Fig. 2G). Additionally, the result of western blot revealed that the expression of cell cycle-related proteins (CDK4, CyclinD1) decreased in PPP1R13L-knockdown SiHa cells but increased in PPP1R13L-overexpressing HeLa cells (Fig. 2H). Cyclin D1/Cdk4 is a key target of the AKT survival signaling pathway.

### PPP1R13L downregulates PTEN to activate the PI3K/AKT/mTOR pathway and upregulates glycolysis in cervical cancer cells

PPP1R13L was first reported to be associated with glycolysis in colon cancer [22]. According to Fig. 1E, PPP1R13L is significantly positively correlated with the cellular response to hypoxia. Therefore, to determine whether glycolysis in cervical cancer cells is associated with PPP1R13L expression, we assessed lactate production and glucose uptake in these cells. The results indicate that the knockdown of PPP1R13L inhibited lactate production and glucose uptake in SiHa cells, whereas overexpression of PPP1R13L enhanced it in HeLa cells (Fig. 3A).

Based on Fig. 1E, PPP1R13L exhibits a significant positive correlation with the PI3K/AKT/mTOR signaling pathway. This pathway facilitates glycolysis by upregulating glycolytic enzymes and augmenting glucose uptake, thereby meeting the metabolic demands of cancerous and rapidly proliferating cells. PTEN acts as a pivotal negative regulator of the AKT/mTOR pathway and exacerbating tumorigenesis and metabolic dysregulation. Consequently, the PTEN/AKT/mTOR pathway plays a crucial role in regulating glycolysis. In light of these, we sought to elucidate the impact of PPP1R13L knockdown and overexpression on the PTEN/AKT/mTOR pathway and its downstream glycolytic enzymes in cervical cancer cells. We observed that PPP1R13L knockdown in SiHa cells resulted in an increase in PTEN protein levels. Concurrently, there were elevated levels of p-AKT/AKT, p-mTOR/mTOR, and HIF1A, as well as increased levels of key glycolytic enzymes (HK2, PGK1, and LDHA). Conversely, PPP1R13L overexpression in HeLa cells led to a decrease in PTEN protein levels, accompanied by reductions in p-AKT/AKT, p-mTOR/mTOR, and HIF1A levels, as well as decreased levels of key glycolytic enzymes (HK2, PGK1, and LDHA) (Fig. 3B). These findings were validated by western blot.

Furthermore, we simultaneously performed PPP1R13L knockdown and overexpression in SiHa and HeLa cells and assessed *PTEN* mRNA expression levels using RT-qPCR. We found that knockdown of PPP1R13L significantly increased *PTEN* mRNA expression levels by 1.5 to 2-fold in both SiHa and HeLa cells. Our results demonstrated that PPP1R13L knockdown significantly elevated *PTEN* mRNA expression levels by 1.5 to 2-fold in both cell lines. In contrast, PPP1R13L overexpression led to a significant reduction in *PTEN* mRNA expression levels (Fig. 3C). ACTB was used the internal control. Additionally, we used western blot to demonstrate the effects of PPP1R13L knockdown and overexpression on the protein levels of PTEN and the AKT/mTOR pathway in SiHa and HeLa cells (Fig. S1D). The above results show that PPP1R13L activates the PTEN/AKT pathway, as well as the glycolytic phenotype and several key glycolytic enzymes.

Given that we have previously discovered that PPP1R13L can induce opposing changes in PTEN levels at the mRNA level (Fig. 3C), we conducted the following experiments to investigate whether PTEN plays a role in the effects of PPP1R13L on cervical cancer cells. For this purpose, we employed PTEN-targeting siRNA and a PTEN overexpression plasmid. We conducted concurrent knockdown of PPP1R13L and PTEN in SiHa cells to counteract the increase in PTEN mRNA and protein levels caused by PPP1R13L knockdown. Conversely, in HeLa cells, we overexpressed PTEN alongside PPP1R13L to offset the decrease in PTEN mRNA and protein levels induced by PPP1R13L overexpression. The CCK-8 and clonogenic assays showed that PTEN knockdown reversed the proliferation inhibition caused by PPP1R13L knockdown in SiHa cells and PTEN overexpression reversed the proliferation-promoting effects of PPP1R13L overexpression in HeLa cells (Fig. S2A, B). To explore whether PTEN is crucial to the effect of PPP1R13L on migration, we performed wound-healing and transwell assays. The results from these assays demonstrated that PTEN knockdown or overexpression reversed the effects of PPP1R13L knockdown or overexpression on the migration of cervical cancer cells (Fig. S2C, D). We then assessed EMT markers (E-cadherin, N-cadherin, ZEB1, Vimentin, Snail, and MMP-2). The results showed that PTEN knockdown reversed the increase in E-cadherin and the decrease in N-cadherin, ZEB1, Vimentin, Snail, and MMP-2 observed with PPP1R13L knockdown in SiHa cells. Correspondingly, PTEN overexpression reversed the changes in these proteins induced by PPP1R13L overexpression in HeLa cells (Fig. S2F). Additionally, we assessed the cell cycle proportion in these transfected cell groups using flow cytometry and examined the levels of cell cycle-related proteins (CyclinD1, CDK4) via western blot. Cyclin D1/Cdk4 is a key target of the AKT survival signaling pathway. We found that PTEN knockdown or overexpression could reverse the changes in cell cycle distribution and the levels of proteins involved in cell cycle regulation caused by PPP1R13L knockdown or overexpression (Fig. S2E, G).

To further elucidate the role of PTEN in modulating the effects of the PPP1R13L/AKT/glycolysis axis, we conducted additional experiments. In SiHa cells, we performed PTEN knockdown during PPP1R13L knockdown, whereas in HeLa cells, we overexpressed PTEN with PPP1R13L overexpression. We subsequently analyzed the expression levels of key glycolytic enzymes in total protein extracts from cervical cancer cells via western blot, alongside quantifying lactate production and glucose uptake. The results indicated that PTEN knockdown reversed the reductions in p-AKT/AKT, p-mTOR/mTOR, and HIF1A levels, as well as the decreased levels of key glycolytic enzymes (HK2, PGK1, and LDHA) caused by PPP1R13L knockdown in SiHa cells. Conversely, PTEN overexpression also reversed the changes in these proteins induced by PPP1R13L overexpression in HeLa cells (Fig. 3D). For the glycolytic phenotype, PTEN knockdown or overexpression reversed the changes in lactate production and glucose uptake induced by PPP1R13L knockdown or overexpression (Fig. 3E).

Subsequently, we established a mouse cervical cancer xenograft model using SiHa cells. PPP1R13L knockdown resulted in reduced tumor growth, with significantly decreased terminal tumor mass and size, demonstrating that PPP1R13L knockdown effectively suppressed the development of SiHa-derived tumors in vivo. (Fig. 3F). The immunohistochemistry (IHC) analysis of tumor xenograft tissues showed that SiHa-shPPP1R13L exhibited a lower Ki67 index and higher PTEN expression, consistent with the results of in vitro experiments (Fig. 3G). Moreover, the expression of EMT marker Snail and the glucose transporter 1 GLUT1 was also reduced in the SiHa-sh-PPP1R13L group. These two proteins are downstream targets of the AKT pathway.

### PPP1R13L inhibits the transcriptional activity of both p53 and TAp63 on PTEN

For the regulation of the tumor suppressor PTEN, p53 functions as a transcription factor, binding directly to its promoter [23]. This interaction leads to elevated levels of PTEN mRNA and protein, thereby contributing to the negative regulation of cellular survival. As we mentioned above, PPP1R13L is one of the most conserved inhibitors of p53 [4]. Based on our findings that PPP1R13L regulates PTEN at the mRNA level, we hypothesize that this effect may be mediated by PPP1R13L’s attenuation of p53-driven transcription of PTEN.

**Fig. 4:**
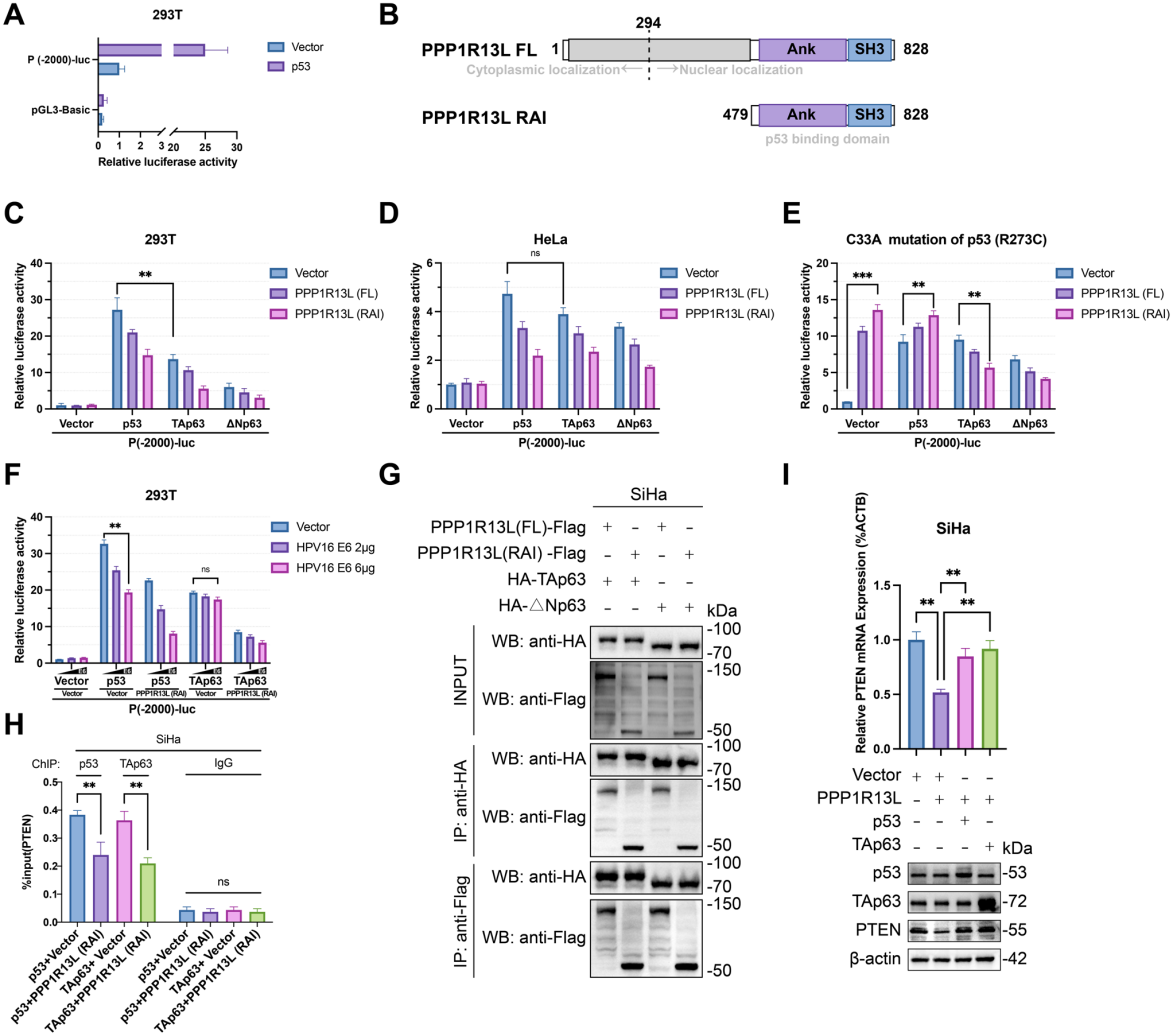
PPP1R13L inhibits the transcriptional activity of both p53 and TAp63 on PTEN. (**A**) 293T cells were transfected with pGL3-basic and P(-2000)-luc, a luciferase reporter containing the PTEN promoter. The results show that P(-2000)-luc has promoter activity and can be enhanced by p53 over 20-fold. (**B**) PPP1R13L full length (FL) consists of 828 amino acids. PPP1R13L (RAI) contains a C-terminal Ank-SH3 domain (amino acids 479–828). (**C**) Assess the activation of P(-2000)-luc by p53 family transcription factors and the inhibition by PPP1R13L (FL) and PPP1R13L (RAI) in 293T cells. The results show that p53 has a significantly higher activation capacity than TAp63 in 293T cells. And PPP1R13L (RAI) exhibits stronger inhibitory activity than PPP1R13L (FL). (**D**) Assess the activation of P(-2000)-luc by p53 family transcription factors and the inhibition by PPP1R13L (FL) and PPP1R13L (RAI) in HeLa cells. In the context of cervical cancer cells where E6 continuously degrades p53, p53 and p63 exhibit comparable activation abilities on P(-2000)-luc. (**E**) The same experiment was repeated in C33A p53 (R273C) cells. The results showed that p53^R273C^ caused PPP1R13L to have an opposite effect, which could not be reversed even with the overexpression of wild-type p53. However, PPP1R13L still inhibited p63’s activation of P(-2000)-luc independently of p53. (**F**) Assess the ability of p53 and p63 to activate the P(-2000)-luc reporter under conditions of gradient transfection of the E6 protein. The results demonstrated that, in the context of continuous degradation of p53 by E6, p63 exhibits transcriptional activity comparable to or even surpassing that of p53 in an E6 dose-dependent manner. Additionally, a synergistic effect between E6 and PPP1R13L (RAI) was observed, resulting in the strongest inhibitory effects on transcription. (**G**) Co-immunoprecipitation and western blot analysis showed that both full-length PPP1R13L and RAI can bind to HA-TAp63 and HA-ΔNp63 in SiHa cells. (**H**) The ChIP assay demonstrated that PPP1R13L (RAI) reduced the binding of both p53 and TAp63 to the PTEN promoter. (**I**) Overexpression of p53 or TAp63 reversed the reduction in PTEN caused by PPP1R13L overexpression, indicating that the regulation of PTEN by PPP1R13L is mediated through p53 or TAp63. *N* = 3 per group. Data are expressed as the mean ± SD. One-way ANOVA assessed multiple-group differences. ns, *p* > 0.05; *p < 0.05; **p < 0.01; ***p < 0.001

**Fig. 5:**
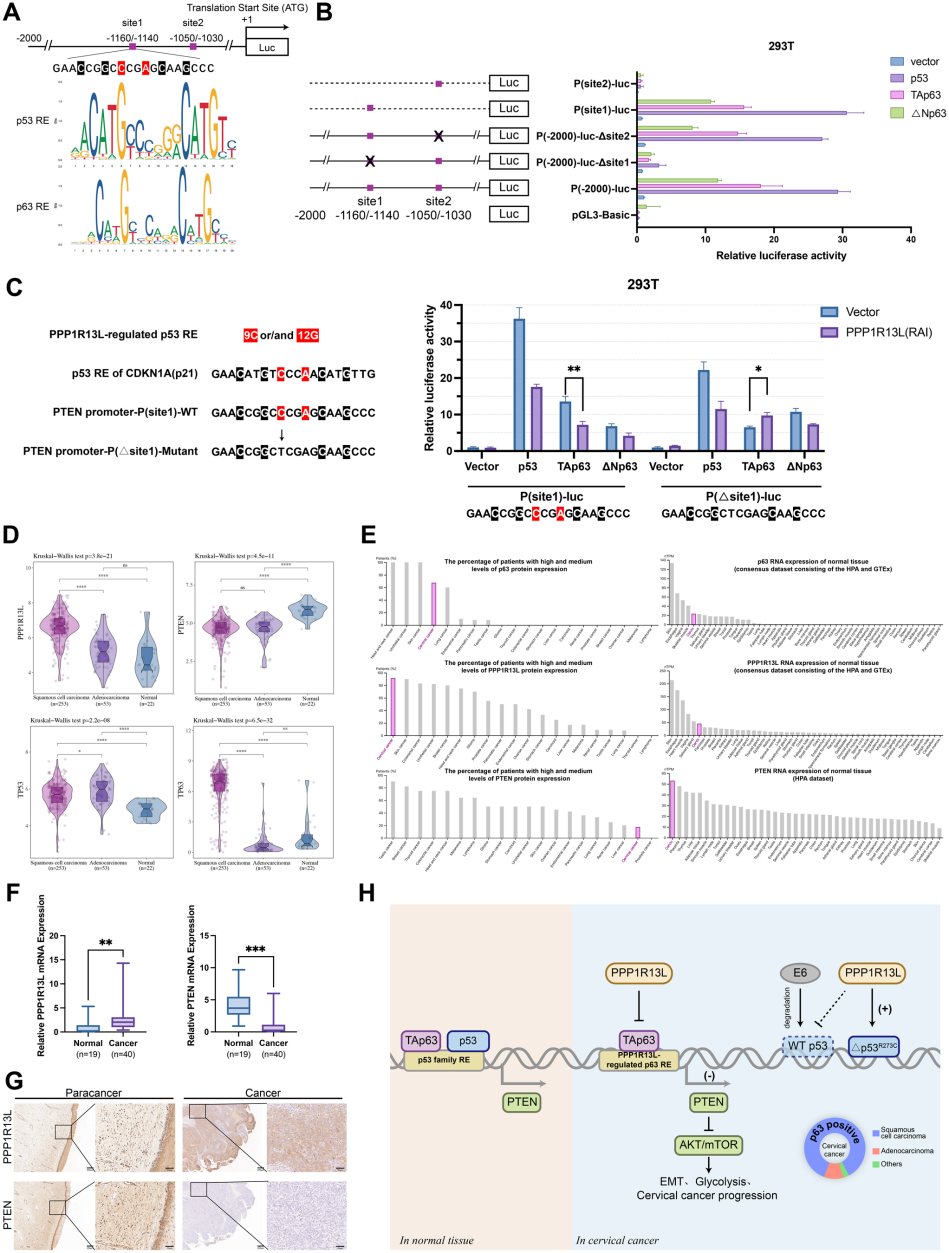
PPP1R13L inhibits the transcription of PTEN by p53 and p63, dependent on specific response elements. (**A**) Two p53 and p63 binding sites were predicted on the PTEN promoter. (**B**) The p53 family proteins were used to activate P(site1)-luc, P(site2)-luc, P(-2000)-luc-△site1, P(-2000)-luc-△site2, P(-2000)-luc, and pGL3-Basic in 293T cells. The dual-luciferase reporter assay showed that site 1 is the active site. (**C**) The PPP1R13L-regulated p53 REs (response element) required the 9 C or/and 12G in the basal p53 REs. The p53 RE in the PTEN promoter is similar to the known PPP1R13L-regulated p53 target gene, p21. The black bases represent the conserved characteristics of p53 RE and the red bases represent the features required for PPP1R13L regulation. The C9T mutation in site1 caused overexpression of PPP1R13L to enhance, rather than inhibit, p63’s transcriptional activity on the PTEN promoter. *N* = 3 per group. Data are expressed as the mean ± SD. One-way ANOVA assessed multiple-group differences. ns, *p* > 0.05; *p < 0.05; **p < 0.01; ***p < 0.001. (**D**) RNA expression of PPP1R13L, PTEN, TP53, and TP63 in cervical squamous cell carcinoma, cervical adenocarcinoma, and normal cervical tissue. Data were obtained from TCGA and GTEx datasets. PPP1R13L and p63 are highly expressed in cervical squamous cell carcinoma but not in cervical adenocarcinoma. (**E**)The positive rate of PPP1R13L, p63, and PTEN in 20 types of tumor slides was ranked, and the RNA expression of them across 55 types of normal tissues were ranked. Data were obtained from The Human Protein Atlas websites. Cervix and cervical cancer have been highlighted in pink. PPP1R13L and p63 are frequently highly expressed in tissues like skin and mucosa, including normal and cancerous cervical tissues. PTEN shows high expression in normal cervical tissues but extremely low positivity in cervical cancer, suggesting it may be suppressed by cervical tissue-specific mechanisms. (**F**) Expression of PPP1R13L and PTEN in cervical cancer tissue (*n* = 40) and normal cervical tissue (*n* = 19). (**G)** IHC results show that adjacent normal tissues with intact cervical mucosa have high PTEN expression, whereas cancer tissues with a loss of mucosal structure exhibit little to no PTEN expression. PPP1R13L expression is positive in adjacent normal tissues but is even higher in cancer tissues. Scale bar represents 50 μm (left) and 200 μm (right). (**H**) Our study shows that PPP1R13L promotes cervical cancer progression, EMT, and glycolysis through the PTEN/AKT/mTOR pathway. Mechanistically, PPP1R13L regulates PTEN transcription via its Ank-SH3 domain interaction with p53 family members, including p53 and p63. Given the low levels of p53 and high levels of p63 in cervical cancer, or in cases of p53 mutation, we focus on the role of the PPP1R13L/p63/PTEN axis in cervical cancer progression

In addition, it has been reported that PPP1R13L weakens p63-mediated transcription, similarly to p53 [20, 21]. As a member of the p53 family, p63 is essential for the development of stratified squamous epithelium and is commonly expressed in basal keratinocytes [18, 19]. In cervical cancer, p63 is used as a marker to differentiate cancer types, with most squamous cell carcinomas showing diffuse nuclear immunoreactivity for p63 [18]. Given p63’s specific role in cervical cancer, we have expanded our research to investigate the effects of PPP1R13L on p63. The p63 gene has two alternative promoters that produce two isoforms: TAp63 and ΔNp63 [24]. TAp63 contains an N-terminal transactivation domain similar to that of p53, while ΔNp63 has a truncated version [25]. TAp63 acts as a tumor suppressor in various cancers, whereas ΔNp63 can function as an oncogene by inhibiting p53, TAp63, and TAp73 [25].

Given that the PTEN has multiple transcription start sites, we designated the translation start site (ATG) as + 1 (NM_000314.8) [26]. We cloned the 2 kb upstream fragment of the translation start site into the luciferase reporter vector pGL3-Basic, naming it P (-2000)-luc, to assess the promoter’s transcriptional activity via luciferase assays. We first co-transfected P (-2000)-luc with either p53 or vector into 293T cells, performing the same procedure with the empty pGL3-basic vector for comparison. Throughout this experiment and subsequent dual-luciferase reporter assays, we consistently co-transfected the pRL-TK plasmid as a normalization control. The results showed that, when normalized to the P (-2000)-luc with vector group, the relative luciferase activity of the pGL3-basic group was very low. In contrast, P (-2000)-luc with p53 overexpression exhibited a relative luciferase activity that was up to 20-fold higher (Fig. 4A).

PPP1R13L, an 828 aa protein, can be cleaved by Caspase-3, producing a stable PPP1R13L (295–828aa) fragment that moves from the cytoplasm to the nucleus. Nuclear translocation is a precondition for its inhibition of p53 transcription, thus this truncated form exhibits a stronger inhibitory effect on p53 compared to the full-length (FL) PPP1R13L [27]. This phenomenon occurs because the N-terminal (1–294aa) of PPP1R13L has a cytoplasmic localization function, whereas the Ank-SH3 domain, which specifically binds to p53, is located at the C-terminal [21]. For PPP1R13L to exert its inhibitory effect on p53, its C-terminal must separate from its N-terminal segment and translocate to the nucleus, a cleavage process facilitated by Caspase-3 [27]. This Caspase cleavage site is conserved across species (from zebrafish to humans), allowing the 295–828 aa fragment to enter the nucleus [27]. Therefore, in HPV-induced cervical cancer, the PPP1R13L fragment (295–828 aa) enters the nucleus and interacts with p53 via its ANK-SH3 domain. In line with this, the nuclear-localized RAI PPP1R13L (479–828aa) was the first form of PPP1R13L identified [4]. The full-length PPP1R13L (828 aa) was discovered a year later and is found in both the cytoplasm and the nucleus, with the N-terminal portion being entirely cytoplasmic (Fig. 4B) [28]. Therefore, we utilized PPP1R13L (RAI) to further explore the effect of PPP1R13L on PTEN mediated by the p53 and p63.

We first transfected P (-2000)-luc into 293T cells, followed by the overexpression of combinations of vector/p53/TAp63/ΔNp63 with vector/PPP1R13L (FL)/PPP1R13L (RAI). According to the results of the dual-luciferase reporter assay, we found that p53, TAp63, and ΔNp63 all enhanced the transcriptional activity of P (-2000)-luc, with the order of potency being p53 > TAp63 > ΔNp63. On this basis, both PPP1R13L (FL) and PPP1R13L (RAI) inhibited the transcriptional activity of P (-2000)-luc induced by the p53 and p63, compared to the vector control. Notably, PPP1R13L (RAI) exhibited stronger inhibitory effects than PPP1R13L (FL), reducing the activity of P (-2000)-luc by approximately 50% (Fig. 4C).

Subsequently, we repeated the experiment in HeLa cells using the same groups. We observed that PPP1R13L (FL) and PPP1R13L (RAI) continued to inhibit the activity of P (-2000)-luc induced by the p53 and p63, with PPP1R13L (RAI) showing stronger inhibition than PPP1R13L (FL). However, although p53 significantly enhanced the activity of P (-2000)-luc more than TAp63 and ΔNp63 in 293T cells, this difference was minimal in HeLa cells (Fig. 4D). The persistent degradation of p53 by E6 protein is one of the most important mechanisms of progression in most cervical cancers [3]. We speculate that the E6 protein of HPV in HeLa cells may keep p53 protein levels low, leading to p53 and TAp63 exhibiting similar transcriptional activity towards the PTEN gene.

Additionally, we repeated the above experiments in an HPV-negative cervical cancer cell line C33A with p53 mutation (R273C) and obtained unexpected results. C33A cells lack wild-type p53 expression, leading to minimal baseline levels of PTEN and PPP1R13L, downstream targets of p53. We found that in the vector and p53 groups, PPP1R13L (FL) and PPP1R13L (RAI) surprisingly promoted the transcriptional activity of P (-2000)-luc, with PPP1R13L (RAI) exhibiting a stronger effect. In contrast, the groups of TAp63 and ΔNp63 maintained the result consistent with those observed in 293T and HeLa cells (Fig. 4E). These findings suggest that the abnormal function of PPP1R13L is due to the p53 mutation (R273C). We observed that PPP1R13L not only exerts an opposite effect in the vector group but also in the p53 group. We speculate this may be because p53 functions as a tetramer in transcriptional regulation, and even overexpression of the wild-type p53 cannot reverse this effect due to the presence of endogenous mutant p53. This is the first time that the opposite role of PPP1R13L has been noted in a tumor cell line with a p53 mutation. As for the p63 groups, the overexpression of PPP1R13L reversed the effects observed in the vector group, suggesting that the effect of PPP1R13L/p63 interaction is much stronger than previously recognized and independently of p53.

To support our hypothesis that when p53 is degraded by the HPV E6 protein, p63 exhibits transcriptional activity comparable to or even surpassing that of p53 in an E6 dose-dependent manner, we conducted gradient overexpression of the HPV16 E6 protein in 293T cells (Fig. 4F). According to our dual-luciferase assay results, in the presence of E6, the transcriptional activity of p53 on PTEN is significantly diminished, whereas the transcriptional activity of p63 on PTEN remains almost unaffected by E6. In the absence of E6, p53’s transcriptional activity on PTEN far exceeds that of TAp63. However, when 6 µg of E6 is transfected, the transcriptional activity of p53 approaches that of TAp63.These results are consistent with observations in HPV-positive HeLa cells. Furthermore, our findings suggest a synergistic effect between E6 and PPP1R13L (RAI) (Fig. 4F). Concurrent overexpression of E6 and PPP1R13L (RAI) results in the strongest inhibitory effects on transcription, although their inhibitory pathways differ: E6 degrades p53, while PPP1R13L does not degrade them but merely inhibits their transcriptional functions.

In 2011, Mario Notari et al. identified the interaction between p63 and PPP1R13L in HaCaT cells and observed that nuclear iASPP colocalized with p63 in human cervical epithelia [20]. Additionally, Deborah J. Robinson in 2019 identified the interaction between PPP1R13L and ΔNp63 [29]. In our study, we overexpressed PPP1R13L (FL)-Flag and PPP1R13L (RAI)-Flag with HA-TAp63 and HA-ΔNp63 in SiHa cells. Using co-immunoprecipitation combined with western blot, we identified the interaction between PPP1R13L and both TAp63 and ΔNp63 proteins in cervical cancer cells (Fig. 4G). This interaction forms the physical basis for PPP1R13L’s inhibition of TAp63 and ΔNp63 transcriptional activity.

Next, we overexpressed vector/p53/TAp63/ΔNp63 in HeLa and SiHa cells and measured *PTEN* mRNA levels. We found that both p53 and TAp63 increased *PTEN* mRNA levels to a similar extent, while ΔNp63 decreased *PTEN* mRNA levels (Fig. S3C). Although ΔNp63 can enhance the transcriptional activity of P (-2000)-luc, it may function as an oncogene by inhibiting others of the p53 family. Under physiological conditions, ΔNp63 ultimately appears to suppress PTEN transcription. Through ChIP experiments, we demonstrated that PPP1R13L weakens the binding of p53 and p63 to the PTEN promoter (Fig. 4H).

Finally, upon overexpressing PPP1R13L, we simultaneously overexpressed p53 or TAp63. This intervention reversed the PPP1R13L-induced reduction in PTEN, indicating that the regulation of PTEN by PPP1R13L is mediated through p53 or TAp63 (Fig. 4I).

These results not only demonstrate that the transcriptional functions of p53 and p63 on PTEN can be inhibited by PPP1R13L, but also highlight the specificity of the p63/PPP1R13L interaction in cervical cancer, particularly when p53 protein levels are low or when p53 is mutated.

### PPP1R13L inhibits the transcription of PTEN by p63, dependent on specific response elements

Next, we aimed to further identify the specific sites on the PTEN promoter where PPP1R13L inhibits the transcription of the p53 family. Using the JASPAR website, we predicted that p53 and p63 have two binding sites within 2 kb upstream of the translation start site, named site1 (-1160/-1140) and site2 (-1050/-1030). Since the response elements (RE) for p53 and p63 are similar, both 2 sites were predicted as potential regions for p53 and p63 binding (Fig. 5A).

We constructed deletion mutants of the P (-2000)-luc plasmid, lacking either site1 or site2, as well as pGL3-Basic plasmids containing only the binding sequence at site1 or site2. We then transfected 293T cells with the empty pGL3-Basic, P (-2000)-luc, P (-2000)-luc-Δsite1, P (-2000)-luc-Δsite2, P(site1)-luc, and P(site2)-luc plasmids, along with vector/p53/TAp63/ΔNp63. The results showed that when normalized to the P (-2000)-luc + vector group, P (-2000)-luc-Δsite2 and P(site1)-luc exhibited promoter activity close to that of the full-length promoter, while P (-2000)-luc-Δsite1 showed only weak promoter activity, and P(site2)-luc exhibited promoter activity similar to that of the empty pGL3-Basic (Fig. 5B). We identified site1 as the region on the PTEN promoter that can be activated by p53, TAp63, and ΔNp63.

PPP1R13L regulates only a subset of p53 target genes, not all. The detailed mechanism for the sequence-specific regulation of p53 by PPP1R13L was first discussed in 2019 by Chen [30]. Most p53 target genes contain a specific DNA sequence, termed a response element (RE). Chen proposed sequence signatures of PPP1R13L-regulated p53 REs, which feature C9 and/or G12 in addition to the typical p53 RE. For example, the p53 target gene p21, regulated by PPP1R13L, has a typical 20-bp p53 RE, characterized by C4, G7, C14, and G17. Simultaneously, its C9 and A12 align with the sequence signatures proposed by Chen [11, 30](Fig. 5C).

We observed that the site1 we identified shares a similar signature with the p53 RE of p21. We mutated C9 to A9 in site1, which deviated from this pattern. We transfected P(site1)-luc and P(△site1)-luc into 293T cells, and co-transfected vector/p53/TAp63/ΔNp63 with PPP1R13L (RAI). We found that P(△site1)-luc still exhibited some promoter activity. In the p53 and ΔNp63 groups, P(△site1)-luc did not alter the inhibitory effect of PPP1R13L (RAI). Notably, the basal transcriptional activity of ΔNp63 increased after the mutation, while the basal transcriptional activities of p53 and TAp63 decreased. Most significantly, in the TAp63 group, the transcriptional activity of P(△site1)-luc was not diminished by PPP1R13L (RAI), but instead exhibited an increase. This observation indicates that site1, characterized by the pattern of C4, G7, C9, A12, C14, and G17, is subject to regulation by the PPP1R13L/TAp63 complex, and its C9 is the key element selectively regulated by PPP1R13L (Fig. 5C).

In summary, we further identified that the PPP1R13L/p53 family regulates PTEN at the upstream region (-1160/-1140) of the translation initiation site (Fig. 5A). Additionally, following the pattern proposed by Chen, we performed a C9A mutation at this site, confirming that C9 is the key element for PPP1R13L to selectively TAp63-mediated PTEN transcription. This is the first experimental validation of the sequence signatures of PPP1R13L-regulated p53 family REs (Fig. 5C).

In our study, we not only focused on the PPP1R13L/p53/PTEN but also investigated the PPP1R13L/p63/PTEN due to the significantly high expression of p63 in cervical squamous cell carcinoma, as shown in the TCGA database (Fig. 5D). In contrast, PTEN is more highly expressed in normal cervix, while p53 shows minimal expression differences between cervical squamous cell carcinoma and adenocarcinoma (Fig. 5D).

Additionally, through the Human Protein Atlas website, we compared the expression of PPP1R13L, PTEN, and p63 in 20 cancer pathology tissue slides and RNA expression across 55 types of normal tissues. We found that both normal and cancer tissues exhibit similar expression patterns for PPP1R13L and p63; they are frequently highly expressed in tissues such as skin and mucosa, including normal cervical and cancer tissues. Notably, PPP1R13L has the highest positivity rate in cervical cancer among the 20 tumor types analyzed (Fig. 5E). Interestingly, PTEN showed the highest expression in normal cervical tissues but exhibited extremely low positivity rates in cervical cancer, ranking just above prostate cancer (Fig. 5E). This difference in PTEN expression across tissue types suggests that PTEN may be strongly suppressed by one or more cervical tissue-specific mechanisms, which could play a critical role in the process of cervical carcinogenesis.

To explore the clinical significance of PPP1R13L and PTEN in cervical cancer, we analyzed mRNA expression in cervical cancer tissues (*n* = 40) and normal cervical tissues (*n* = 19) from the Affiliated Zhongnan Hospital of Wuhan University using RT-qPCR. *PPP1R13L* mRNA expression was significantly higher in cervical cancer tissues, while *PTEN* mRNA showed the opposite pattern (Fig. 5F). Furthermore, IHC results from the identical patient show that adjacent normal tissues with intact cervical mucosa have high PTEN expression, while cancer tissues with a loss of mucosal structure exhibit little to no PTEN expression. PPP1R13L is positively expressed in adjacent normal tissues but is even higher in cancer tissues (Fig. 5G).

## Discussion

PPP1R13L regulates cell proliferation, apoptosis, and other cellular behaviors by interacting with p53 and NF-κB proteins [5, 11–15, 31]. As mentioned above, PPP1R13L is highly expressed in tissues such as the skin, esophagus, myocardium, vagina, and cervix (Fig. 5E). In tumors, it has been associated with the progression of cutaneous squamous cell carcinoma and head and neck squamous cell carcinoma [29, 32]. PPP1R13L also shows significant differences in expression between tumor and normal tissues in cancers such as cervical cancer, cholangiocarcinoma, and bladder cancer. In 2017, PPP1R13L was found to induce EMT and cisplatin resistance in cervical cancer [9]. Furthermore, elevated levels of PPP1R13L are associated with poor prognosis in both cervical squamous cell carcinoma and cervical adenocarcinoma [33–35].

Given this context, PPP1R13L in cervical cancer holds particular significance. Our research has revealed that PPP1R13L promotes cervical cancer cell proliferation, EMT, cell cycle and migration by inhibiting PTEN transcription and subsequently activating the AKT pathway. Furthermore, as a major regulatory pathway of metabolism, AKT pathway activation enhances glycolysis in cervical cancer cells. Through rescue experiments, we demonstrated that the reduction of PTEN is an indispensable factor in the progression of cervical cancer induced by PPP1R13L.

Next, we further investigate the mechanism by which PPP1R13L suppresses PTEN transcription through p53 family proteins. PPP1R13L, also known as iASPP, shares high sequence homology with the other two members of the ASPP family of proteins, ASPP1 and ASPP2 in the C-terminal region, which includes the Ank-SH3 domain. This domain mediates the interactions between ASPP family proteins and p53 family proteins. However, PPP1R13L exerts the opposite effect compared to ASPP1 and ASPP2, inhibiting p53 function while ASPP1 and ASPP2 enhance it [21]. This difference is due to subtle variations in the Ank-SH3 domain sequence, where fine-tuning of the interaction interface leads to opposite function. Therefore, our study utilizes not only full-length PPP1R13L but also PPP1R13L (RAI), which includes the Ank-SH3 domain, to determine that PPP1R13L regulates the transcription of PTEN through its Ank-SH3 domain interaction with p53 family proteins, p53 and p63 (Fig. 4C, D, F, I).

In research on the interactions between PPP1R13L and p53 family proteins, p53 is the most studied, while p63 has been less explored. In vitro, PPP1R13L has the highest affinity for p53, and its binding affinity for p63 is three times that for p73 [21]. In 2011, it was the first to confirm that PPP1R13L can bind to and regulate p63 transcription in stratified epithelium in vivo, independent of p53 [20]. p63 expressed in the basal layer of stratified squamous epithelium. It serves as a marker for squamous cell carcinoma. Given that 85–95% of cervical cancers are squamous cell carcinomas, p63 is crucial for diagnosing cervical cancer. It helps differentiate between reactive cervicitis and cervical intraepithelial changes, and is valuable for grading CIN lesions and distinguishing between squamous cell carcinoma, adenocarcinoma, and small cell carcinoma [18, 19, 36]. In 2008, Ross Alexander Robinson and colleagues investigated the interaction between the ANK-SH3 domain of PPP1R13L (iASPP) and the DNA-binding domains of p53, p63, and p73 using a solid-phase binding assay in vitro, evaluating their binding affinities [21]. Since then, discussions about PPP1R13L’s inhibition of the p53 family often mention p53, p63, and p73 together, although research on p73 in this context has been relatively limited. A 2015 melanoma study found that PPP1R13L’s effect on p73 function is primarily evident under cisplatin treatment, with minimal impact in its absence [37]. Due to the scarcity of prior research on p73, the study did not investigate p73 in parallel. However, in non-cervical cancers or non-tumor diseases, the interaction between PPP1R13L and p73 may play a significant role.

Our findings indicate that p53, TAp63, and ΔNp63 all enhance PTEN promoter activity, but only p53 and TAp63 ultimately increase PTEN levels in cervical cancer cells. Furthermore, when comparing results from 293T and HeLa cells, we observed that TAp63’s function in PTEN transcription is more similar to that of p53 in HeLa cells (Fig. 4D). We suggest that this result from the persistent degradation of p53 induced by HPV E6 protein (Fig. 4F). Our results emphasize the critical importance of the PPP1R13L/p63/PTEN in cervical cancer cells, where p63 is highly expressed and p53 levels are diminished.

Surprisingly, in the p53-mutant cell line C33A, we found that the R273C mutation in p53 causes PPP1R13L to function oppositely to wild-type p53, while the effect of p63 remains unchanged (Fig. 4E). This suggests that the R273C mutation in p53 may change the interaction pattern between PPP1R13L and p53. Additionally, the R273C mutation in p53 may alter the interaction pattern between PPP1R13L and p53, causing iASPP’s function to shift to resemble that of ASPP1 and ASPP2. This indicates that the p53 R273C mutation plays a crucial and subtle role in the structural basis for interactions with the ASPP family. A single amino acid mutation in p53 reverses the function of iASPP. Our study is the first to demonstrate that PPP1R13L exerts an opposing effect on p53 in p53-mutant cell lines compared to the wild-type. Our findings also suggest that the PPP1R13L/p63 axis functions independently of p53, as demonstrated by Mario Notari et al. [20]. Therefore, when p53 is mutated, the PPP1R13L/p63 should be a focus of attention. p53 and p63 are structurally and functionally similar “sibling” proteins that can either collaborate or antagonize each other, depending on their isoforms and the cellular environment [38]. In 293T and HeLa cells, the presence of wild-type (WT) p53 makes it difficult to determine whether p63 functions to some extent (even if very weakly) in a p53-dependent manner. In contrast, C33A cells allow us to study p63 independently of p53.

The transcriptional regulation of PTEN by p53 was first described by V. Stambolic, who identified a p53 binding site on the PTEN promoter that is close to the site1 identified in our study [23]. However, they reported that the PTEN promoter contains a p53 binding site with a 14 bp spacer between the two half-sites. Our findings demonstrate that even when only the left half-site, corresponding to site1 in our study, is present, it can effectively activate the PTEN promoter (Fig. 5B). Interestingly, the effect of p63 on PTEN varies across different studies. ΔNp63, considered an oncogene, was reported to repress PTEN expression by reducing PTEN promoter activity through p73-dependent mechanisms in squamous carcinomas of the head and neck [39]. However, Hao T et al. proposed that ΔNp63 enhances PTEN promoter activity, thereby promoting PTEN expression in oral cancer cells [40]. In our study, we found that ΔNp63 only enhances PTEN promoter activity but ultimately inhibits PTEN expression in cervical cancer cells, thereby maintaining its role as an oncogene. Additionally, the two p53 family binding sites predicted by the JASPAR database align with the findings of Hao T et al. [40].

PPP1R13L selectively regulates only a subset of p53 transcriptional targets. The p53 target genes identified as being regulated by PPP1R13L, including BAX, AEN, FAS, FHL2, CDKN1A (p21), PMAIP1, RAP2B, and TIGAR, are primarily involved in the regulation of apoptosis [30]. Chen described sequence signatures enriched in PPP1R13L-regulated p53 REs, providing a possible sequence basis for PPP1R13L-regulated p53 binding [30]. Our study extends these findings to the PPP1R13L/TAp63, confirming the applicability of Chen’s rules (Fig. 5C). As for p53, the presence of only the left half-site in site1 may account for the negative results observed.

In summary, the results show that PPP1R13L promotes cervical cancer cell proliferation, EMT, cycle progression, and glycolysis through the PTEN/AKT/mTOR pathway. Mechanistically, PPP1R13L regulates the transcription of PTEN via its Ank-SH3 domain interaction with p53 and p63(Fig. 5H). Further studies have highlighted three key findings:

1. In cervical cancer cells, sustained degradation of p53 and elevated levels of p63 enable p63 to demonstrate a capacity to regulate PTEN comparable to, or even exceeding, that of p53, depending on the level of HPV E6. Combined with the high expression of p63 and PPP1R13L in cervical cancer, this underscores the importance and uniqueness of the PPP1R13L/p63/PTEN axis in cervical cancer.
2. For the first time, it was discovered that in C33A, an HPV-negative cervical cancer cell line, the △p53^R273C^ mutation causes PPP1R13L to exert an opposite effect on mutant p53. This suggests that the PPP1R13L/p63 functions independently of p53.
3. The response elements of PPP1R13L-regulated p53 family target genes were experimentally validated on p63 for the first instance. This provides the sequence basis for PPP1R13L to selectively regulate p53 family target genes.

## Materials and methods

### Human cervical cancer tissue specimens

This study collected 40 pathologically confirmed cervical cancer tissue specimens and 19 normal cervical tissue specimens from recent patients at the Affiliated Zhongnan Hospital of Wuhan University. The acquisition of all human samples was approved by the Medical Ethics Committee of Zhongnan Hospital of Wuhan University (Ethics Approval No. 2020029), and written informed consent was obtained from all patients or their relatives. All human experiments in this study strictly adhered to the principles of the Declaration of Helsinki.

### Data source and data processing

The RNA expression and related clinical data were obtained from the CESC dataset of TCGA, and the latest version (V8) of the GTEx dataset was acquired through the GTEx data portal. Gene Set Variation Analysis (GSVA) was performed using the GSVA package in R software, with the method parameter set to ‘ssgsea’. Spearman correlation analysis was used to assess the correlation between gene expression and pathway scores. A p-value of < 0.05 was considered statistically significant. The bar charts of high expression rates in 20 cancer pathology tissue sections and the RNA expression levels in 55 types of normal tissues were downloaded from The Human Protein Atlas( https://www.proteinatlas.org/). Several GSE datasets were obtained from GEO, and box plots were generated using R software. The statistical methods employed are specified in figures.

### Cell culture and and transfection

HeLa and SiHa cell lines were cultured in DMEM (Procell, China) medium with 1% penicillin-streptomycin and 10% FBS (Gibco, MD, USA) under 5% CO2 at 37 °C, while C33A cells were cultured in MEM instead of DMEM, with all other conditions remaining the same. Three siRNAs targeting PPP1R13L (si-PPP1R13L#1: 5′‐CCAACUACUCUAUCGUGGAUU‐3′, si‐PPP1R13L#2: 5′‐GCCUCAAAGGAGUAAAGUCUA‐3′, si‐PPP1R13L#3: 5′‐CCCUACCCACAAGAAACAGUA‐3′) and one siRNA targeting PTEN (si‐PTEN: 5′‐CUAGAACUUAUCAAACCCUUU‐3′) were designed and synthesized by Wuhan Tsingke Biotechnology (TSINGKE, China). The pcDNA3.1-vector, pcDNA3.1-PPP1R13L-Full length-FLAG plasmid, pcDNA3.1-PPP1R13L-Ank-SH3-FLAG plasmid, pcDNA3.1-p53 plasmid, pcDNA3.1-Tap63-HA plasmid, pcDNA3.1-△Np63-HA plasmid and pcDNA3.1-PTEN plasmid were purchased from Miaoling Biology (Wuhan, China). The transfection was performed using Lipofectamine 2000 (Invitrogen, USA) or PEI (Polyethylenimine) (YEASEN, China). Follow-up experiments were conducted 24 to 72 h post-transfection.

### Cell migration assays

3 × 10⁴ cells in 100 µl medium without FBS were plated onto a transwell chamber (Corning, USA). As a chemoattractant, 500 µl medium (DMEM with 10% FBS) was added to the lower chamber. After a 48-hour, the cells that adhered to the lower surface of the membrane were stained with gentian violet and counted under a microscope across five predefined fields at 100× magnification. Each assay was performed independently at least three times.

### Wound-healing assay

SiHa and HeLa cells were seeded into 6-well plates. And linear wounds were created with a sterile 200 µL pipette tip when the cell confluence reached 90%. Wound closure was imaged with an inverted microscope at 0, 24, 48 h post-wounding. The average distance between the two opposing edges of the cell-free area was used to determine the wound healing rate.

### Assessment of glycolytic phenotype

Glucose consumption and lactate production were analyzed using a glucose assay kit (glucose oxidase method, Jiancheng Bio, CHN) and a lactic acid assay kit (KeyGEN Bio, CHN), respectively, following the manufacturers’ instructions. The results were normalized to the number of cells.

### Cell proliferation and clonogenic assays

2 × 10³ cervical cancer cells in 200 µl were cultured in a 96-well plate, with measurements taken at 1-day intervals using the CCK-8 assay (Servicebio, China). For the clonogenic assay, cells in 6-well culture plates were incubated at 37 °C for 15 days, with the medium changed every 3 days, at an initial density of 300 cells per well. The cells were fixed with 4% paraformaldehyde for 25 min and stained with 0.1% crystal violet.

### Western blot

RIPA lysis buffer (Biosharp, China) with added PMSF and phosphatase inhibitors was used to lyse the cells, following the protocol provided in the manual. The lysates were centrifuged to collect the supernatant, and proteins were denatured in a 100 °C water bath. Proteins were then separated by SDS-PAGE and transferred to PVDF membranes (Millipore, USA). The membranes were blocked with 5% skimmed milk at room temperature for 2 h and incubated overnight at 4 °C with primary antibodies. Afterward, they were incubated with the corresponding secondary antibodies. Following primary antibody incubation, membranes were exposed to the appropriate secondary antibodies. Protein detection was carried out using an ECL chemiluminescence detection kit (ABclonal Biotechnology, China), and protein levels were quantified through grayscale analysis with ImageJ. The antibodies used in this study are listed in Supplementary Table 1.

### RT–qPCR

Total RNA was extracted using RNA extraction reagent (Servicebio, China), and its concentration and quality were checked with a Nanodrop (Thermo, USA). Using the Hiscript II Q Select RT kit and SYBR Green from Vazyme (China), RNA was reverse transcribed and quantified by PCR. This study used the following primers: ACTB-Forward: CATGTACGTTGCTATCCAGGC and ACTB-Reverse: CTCCTTAATGTCACGCACGAT; PPP1R13L-Forward: GAAATCACTGGGGACAGGAA and PPP1R13L-Reverse: CCCAGGAATATCCAGTGGTG; PTEN-Forward: ATGTGGCGGGACTCTTTAT and PTEN-Reverse: GGCTCAACTCTCAAACTTCC. The relative mRNA expression levels were calculated using the 2^-ΔΔCT method, with β-Actin serving as the internal control.

### Dual-luciferase reporter assay

The full-length PTEN promoter, along with site1, site2, △site1-promoter of PTEN, △site2-promoter of PTEN, and △site1, were cloned into the pGL3-Basic vector containing the firefly luciferase reporter gene. These plasmids, as well as the pGL3-Basic vector, were obtained from Miaoling Biology (Wuhan, China). The aforementioned promoter-related pGL3-Basic plasmids, along with overexpression plasmids of the p53 family proteins (p53, TAp63, △Np63), and overexpression plasmids of PPP1R13L (full-length and Ank-SH3), were co-transfected into cervical cancer cells. The pRL-TK plasmid was co-transfected to serve as a normalization control. After 24 h, luciferase activity was measured with the Dual Luciferase Reporter Assay System (RGRG027, ABclonal, China). All experiments were done in triplicate.

### Cell cycle analysis by flow cytometer (FCM)

The cells, transfected for 48–72 h, were harvested by trypsinization without EDTA and washed twice with ice-cold PBS. The cell pellets were resuspended in 1 mL of DNA staining solution along with 10 µL of permeabilization solution (Multi Sciences, CHN) and kept in the dark for 30 min. The samples were then analyzed by flow cytometry (Beckman Coulter) to assess the cell cycle.

### Tumor xenograft model

12 female BALB/C nude mice, aged 4–6 weeks and weighing between 18 and 20 g, were kept at the Animal Testing Center of Central South Hospital of Wuhan University, situated in Vital River, Beijing, China. After a one-week acclimatization period, 12 nude mice were subcutaneously injected with SiHa cells stably transfected with either sh-PPP1R13L or sh-NC lentivirus (1 × 10^7 cells in 100 µL of DMEM) into the axillary region. Starting one week after injection, mouse body weight and tumor size were measured every three days. At the fourth week post-injection, mice were euthanized at the cervical region and tissues were harvested. Tumor volume and weight were measured, and the specimens were fixed in 4% paraformaldehyde. The formula for calculating tumor volume is: Volume = (length × width^2) / 2. The animal study received approval from the Experimental Animal Ethics Committee of Zhongnan Hospital of Wuhan University (approval number: 02523064a).

### Immunohistochemistry

The cervical cancer slides were incubated at 60 °C for 2 h, deparaffinized with xylene, and rehydrated using graded ethanol. Citrate buffer was used for antigen retrieval, and endogenous peroxidase activity was blocked with 3% hydrogen peroxide. The slides were kept overnight at 4 °C with the first antibodies after BSA blocking, then with secondary antibodies. Staining was performed with xylene and hematoxylin, and the slides were sealed with neutral gum for analysis under a fluorescence microscope.

### Chromatin immunoprecipitation (ChIP) assay

4 × 10^6^ SiHa cells were prepared for the ChIP assay using a kit from Beyotime (China). Ultrasonic treatment was used to shear genomic DNA after 1% formaldehyde crosslinking of cells. 3 µg of p53 antibody (ABclonal, A19585), 3 µg of p63 antibody (ABclonal, A26170), or IgG was added to the respective samples. Subsequently, 70 µl of protein A + G Agarose/Salmon Sperm DNA was added for immunoprecipitation to obtain the DNA fragments bound to p53 or p63. Afterward, DNA extraction and purification were performed. After the reversal of cross-linking, the DNA was purified and analyzed by qPCR to amplify a 152-bp region that includes site 1 of the PTEN promoter. The primer is ‘Forward: GTTATCCTCGCCTCGCGTTG, Reverse: GTAGCTCTGGGTGCGAGC’.

### Statistical analysis

All experiments were independently repeated at least three times. Statistical analysis was conducted using SPSS software and GraphPad Prism. Group differences were evaluated using either one-way ANOVA or a two-tailed Student’s t-test, depending on the context. A p-value of less than 0.05 was considered statistically significant.

## Electronic Supplementary Material

Below is the link to the electronic supplementary material.

Supplementary Material 1

## Data Availability

Not applicable.

## Abbreviations

siRNA: small interfering RNA
shRNA: short hairpin RNA
PIP3: phosphatidylinositol (3,4,5)-trisphosphate
PIP2: phosphatidylinositol (4,5)-bisphosphate
CCK8: Cell Counting Kit-8
PTEN: phosphatase and tensin homolog
GSEA: single-sample gene set enrichment analysis
RAI: RelA-associated inhibitor
HK2: hexokinase 2
PGK1: phosphoglycerate kinase 1
LDHA: lactate dehydrogenase A
HIF1A: hypoxia-inducible factor 1-alpha
MMP-2: matrix metalloproteinase-2
ZEB1: zinc finger E-box-binding homeobox 1
CESC: cervical squamous cell carcinoma
TCGA: The Cancer Genome Atlas
EMT: epithelial-mesenchymal transition

## Declarations

### Cell lines

The human cervical cancer cell lines HeLa (CL-0101), SiHa (CL-0210), C33A (CL-0045), and the human embryonic kidney cell line 293T (CL-0005) used in this study were purchased from Procell, Wuhan, China, and were verified by STR profiling.
