# Supplementary material for: PPP1R13L drives cervical cancer progression by suppressing p63-mediated PTEN transcription
Source: Cell Mol Life Sci. 2025 Feb 27;82(1):97. doi: 10.1007/s00018-025-05598-9 (PMC11868476; doi:10.1007/s00018-025-05598-9)
Supplement: Supplementary file 1 — Supplementary Material 1 [file 18_2025_5598_MOESM2_ESM.docx]

**Supplementary material**

**Supplementary Figures:**

**
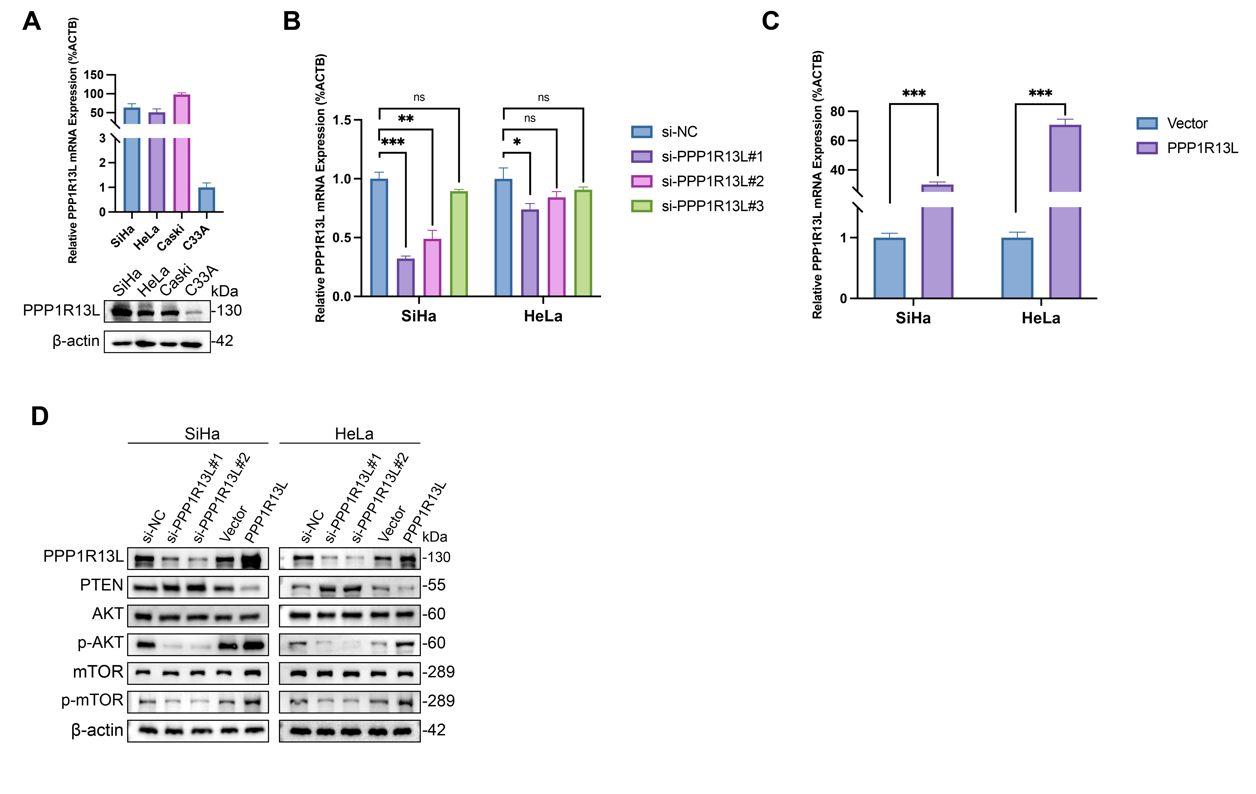
**

**Figure S1**

**The expression of PPP1R13L in cervical cancer and the knockdown or overexpression of PPP1R13L in SiHa and HeLa cells.**

(A) The expression of PPP1R13L in cervical cancer: PPP1R13L is expressed at the highest protein level in SiHa cells. In contrast, C33A, a p53-mutant cervical cancer cell line, shows extremely low PPP1R13L expression, as PPP1R13L is a downstream gene of p53.

(B) PPP1R13L knockdown in SiHa and HeLa cells by siRNA shows that SiHa cells exhibited higher knockdown efficiency.

(C) Overexpression of PPP1R13L in SiHa and HeLa cells shows higher efficiency in HeLa cells.

N = 3 per group. Data are expressed as the mean ± SD. One-way ANOVA assessed multiple-group differences.

(D) Overexpression and knockdown of PPP1R13L were performed in SiHa and HeLa cells, followed by western blot analysis to demonstrate that PPP1R13L regulates the PTEN/AKT/mTOR signaling pathway in cervical cancer cells. ns, *p* > 0.05; **p* < 0.05; ***p* < 0.01; ****p* < 0.001.


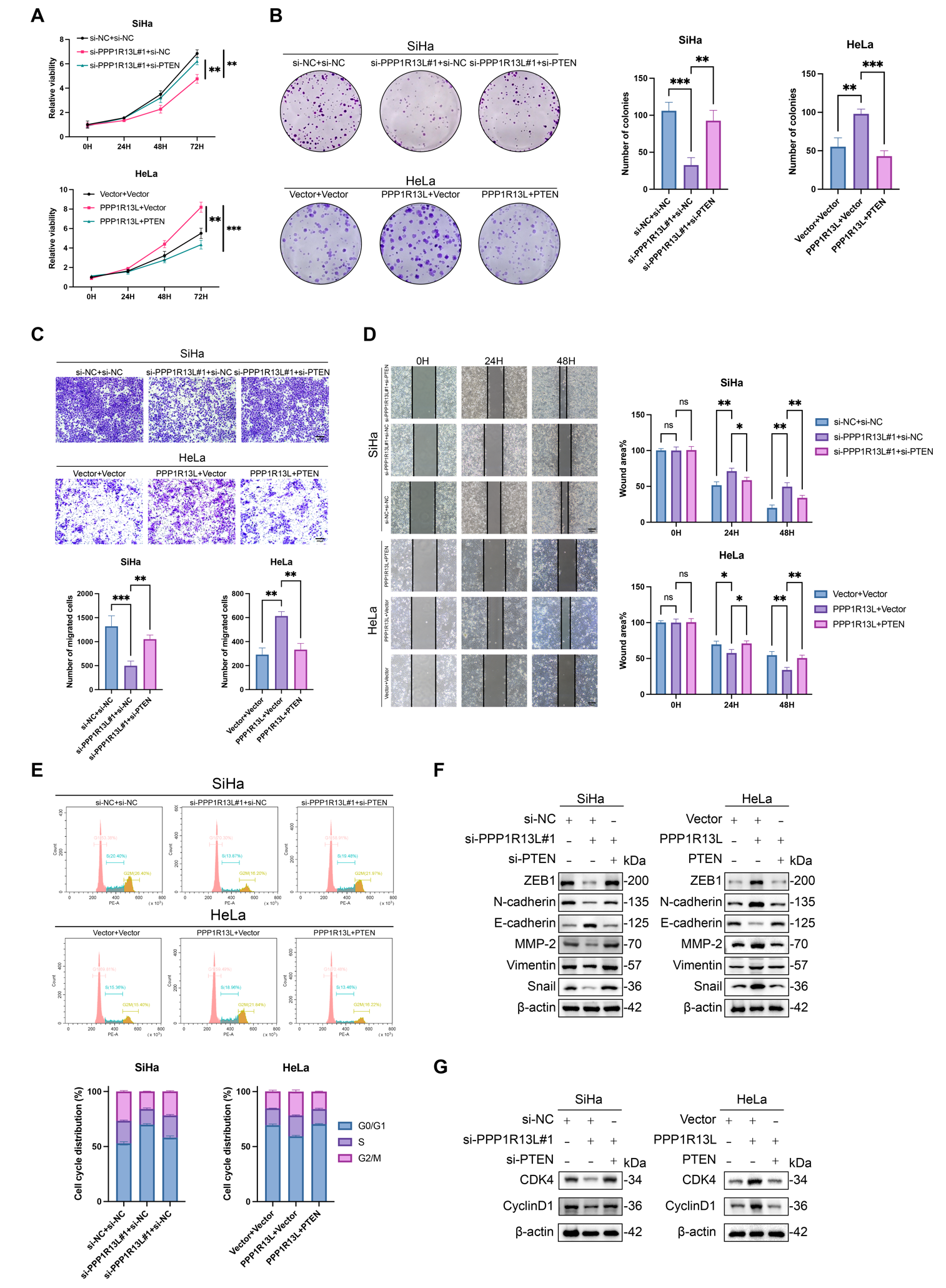


**Figure S2**

**PPP1R13L promotes cervical cancer cell proliferation, cycle progression, and facilitates EMT by downregulating PTEN.**

(A, B) The CCK-8 and colony formation assay revealed that PTEN knockdown reversed the proliferation inhibition caused by PPP1R13L knockdown in SiHa cells and PTEN expression reversed the proliferation-promoting effects of PPP1R13L expression in HeLa cells.

(C, D) Transwell and wound-healing assays revealed that PTEN knockdown reversed the migration inhibition caused by PPP1R13L knockdown in SiHa cells, and PTEN expression reversed the migration-promoting effects of PPP1R13L expression in HeLa cells. Scale bar represents 50 µm.

(E) The cell cycle distribution was detected by flow cytometry. PTEN knockdown reverses the cell cycle arrest in the G0/G1 phase in PPP1R13L-knockdown SiHa cells, while PTEN overexpression reverses cell cycle distribution in PPP1R13L-overexpressing HeLa cells.

(F) Western blot analysis shows that the expression of EMT markers (ZEB1, N-cadherin, E-cadherin, Vimentin, Snail, MMP-2) was reversed by PTEN knockdown in PPP1R13L-knockdown SiHa cells and by PTEN overexpression in PPP1R13L-overexpressing HeLa cells.

(G) Western blot results show that the expression of cell cycle-related proteins is reversed by PTEN knockdown in PPP1R13L-knockdown SiHa cells and by PTEN overexpression in PPP1R13L-overexpressing HeLa cells.

N = 3 per group. Data are expressed as the mean ± SD. One-way ANOVA assessed multiple-group differences. ns, *p* > 0.05; **p* < 0.05; ***p* < 0.01; ****p* < 0.001.


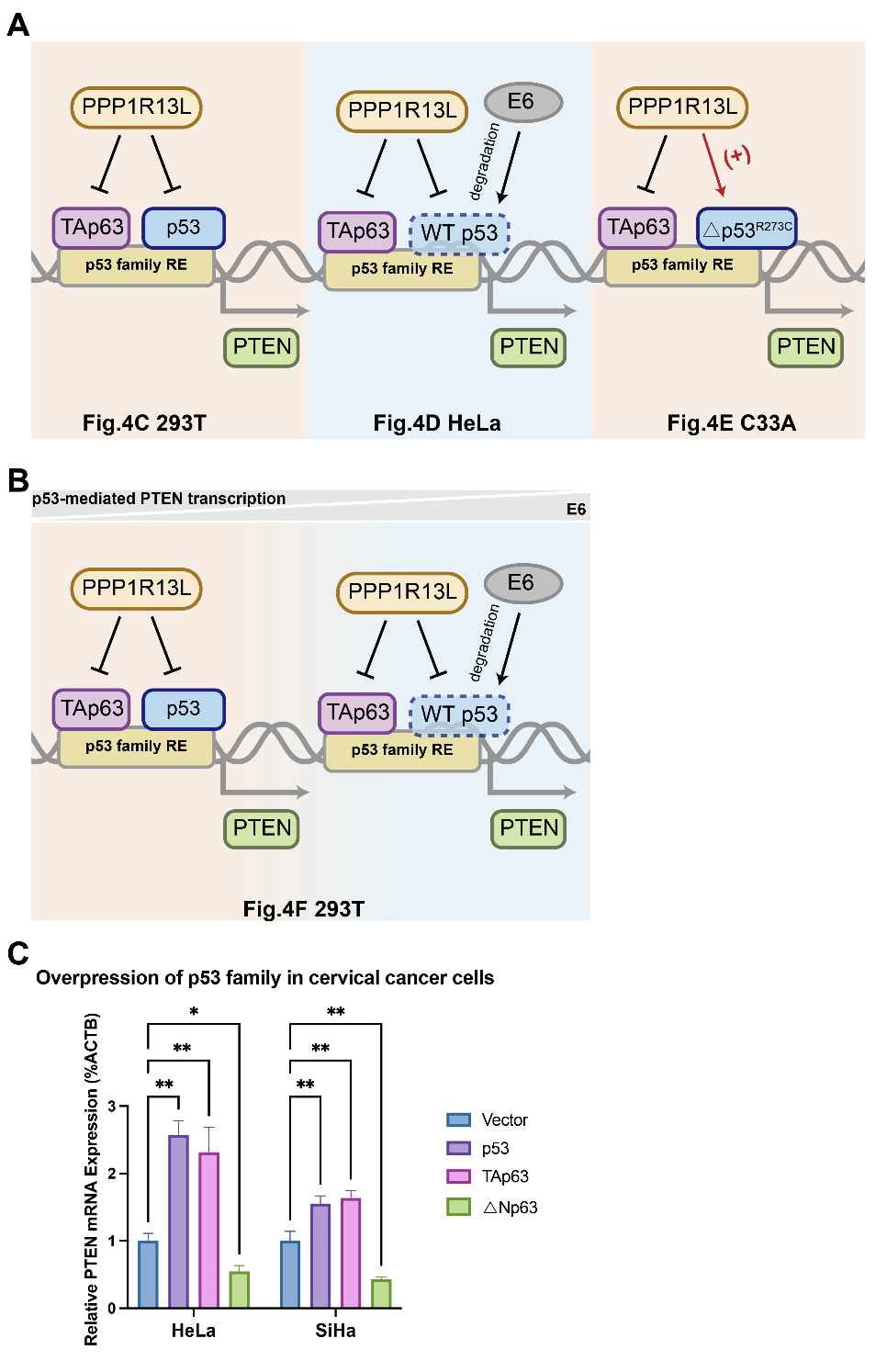


**Figure S3**

**Schematic representation of three parallel experiments in Fig. 4C-E and overexpression of p53 and p63 in SiHa and HeLa cells**

(A) Schematic diagram of the results from three parallel experiments in different cell lines shown in Fig. 4C-E.

(B) Schematic diagram of the results from Fig. 4F.

(C) Overexpression of p53 and TAp63 increased the *PTEN* mRNA in HeLa and SiHa cells, whereas ΔNp63 had the opposite effect. N = 3 per group. Data are expressed as the mean ± SD. One-way ANOVA assessed multiple-group differences. ns, *p* > 0.05; **p* < 0.05; ***p* < 0.01; ****p* < 0.001.


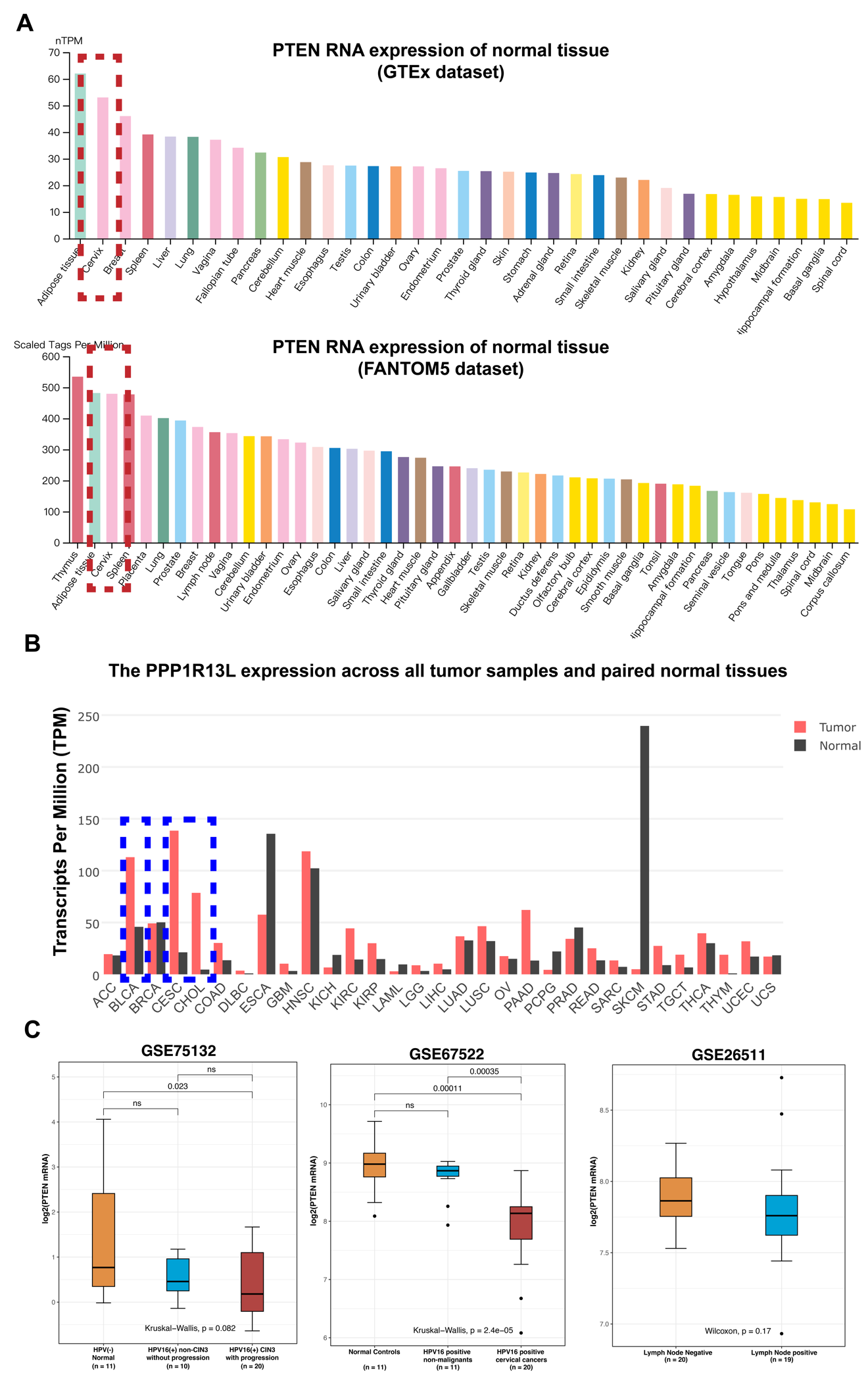
**Figure S4**

**PTEN is highly expressed in normal cervical tissue and PPP1R13L shows differential expression between cancerous and normal tissues in cervical cancer, cholangiocarcinoma, and bladder cancer.**

(A) *PTEN* mRNA expression levels across 55 normal tissues were ranked using data from The Human Protein Atlas, GTEx and FANTOM5 dataset. The cervix and cervical cancer are highlighted with a red box.

(B) The *PPP1R13L* mRNA expression data across all tumor samples and paired normal tissues were obtained from the GEPIA website. *PPP1R13L* mRNA shows expression differences between tumor and normal tissues in cancers such as cervical cancer, cholangiocarcinoma, and bladder cancer.

(C) In the GSE75132 dataset, *PTEN* mRNA exhibits a trend opposite to that of *PPP1R13L* mRNA (consistent with the dataset used in Fig. 1C). Additionally, in the GSE67522 dataset, *PTEN* mRNA shows a decreasing trend across groups, from normal tissues to HPV16 non-malignant tissues to HPV16 malignant tissues. In the GSE26511 dataset (consistent with Fig. 1B), *PTEN* mRNA expression is slightly lower in the lymph node metastasis-positive group compared to the negative group, showing a trend opposite to that of PPP1R13L; however, the difference is not statistically significant.

**Supplementary Table 1: Antibodies used in Western Blot and other experiments.**

| Proteins | Antibody Type and Cat. No. | Reagent company | Dilution ratio |
| --- | --- | --- | --- |
| β-actin | Rabbit mAb, AC026 | ABclonal | 1:10000 |
| PTEN | Rabbit pAb, A11193 | Abclonal | 1:1000 for WB, 1:200 for IHC |
| PPP1R13L | Rabbit pAb, A11194 | Proteintech | 1:1000 for WB, 1:200 for IHC |
| E-Cadherin | Rabbit mAb, A22850 | Abclonal | 1:1000 |
| N-Cadherin | Rabbit pAb, 22018-1-AP | Proteintech | 1:1000 |
| Snail | Rabbit pAb, A11794 | ABclonal | 1:1000 |
| Vimentin | Rabbit mAb, A19607 | ABclonal | 1:1000 |
| ZEB1 | Rabbit pAb, 21544-1-AP | Proteintech | 1:1000 |
| MMP2 | Rabbit pAb, 10373-2-AP | Proteintech | 1:1000 |
| AKT | Rabbit mAb, A18675 | Abclonal | 1:1000 |
| p-AKT | Rabbit mAb, AP1208 | Abclonal | 1:1000 |
| mTOR | Rabbit pAb, 20657-1-AP | Proteintech | 1:1000 |
| p-mTOR | Mouse mAb, 67778-1-Ig | Proteintech | 1:1000 |
| HIF1A | Mouse mAb, ab1 | Abcam | 1:1000 |
| HK2 | Rabbit mAb, A20829 | Abclonal | 1:1000 |
| PGK1 | Rabbit mAb, A12686 | Abclonal | 1:1000 |
| LDHA | Rabbit mAb, A21893 | Abclonal | 1:1000 |
| CDK1 | Rabbit mAb, ab133327 | Abcam | 1:1000 |
| CDK4 | Rabbit pAb, A21317 | ABclonal | 1:1000 |
| Cyclin B1 | Rabbit pAb, 55004-1-AP | Proteintech | 1:1000 |
| Cyclin D1 | Mouse mAb, 60186-1-Ig | Proteintech | 1:1000 |
| HA | Rabbit pAb, 51064-2-AP | Proteintech | 1:3000 for WB, 1ug for CO-IP |
| Flag | Rabbit pAb, 20543-1-AP | Proteintech | 1:3000 for WB, 1ug for CO-IP |
| HRP-conjugated Affinipure Goat Anti-Mouse IgG(H+L) | SA00001-1 | Proteintech | 1:10000 |
| HRP-conjugated Affinipure Goat Anti-Rabbit IgG(H+L) | SA00001-2 | Proteintech | 1:10000 |
